# Bubble formation, growth and rise in non-Newtonian xanthan gum

**DOI:** 10.1039/d6ra03620a

**Published:** 2026-08-14

**Authors:** Md Ishtiaque Hossain, Md. Tariqul Islam, Mohamed Murshid Shamsuddeen, Asif Afzal

**Affiliations:** a Department of Mechanical and Production Engineering, Ahsanullah University of Science and Technology Dhaka 1208 Bangladesh ishti.me@aust.edu; b Clariant Pty Ltd Lara 3212 VIC Australia; c NineplusIT, Corporate R&D Center Seoul South Korea; d University Centre for Research & Development, Department of Computer Science and Engineering, Chandigarh University Gharuan Mohali Punjab India

## Abstract

Xanthan gum (XG) assists in modifying the rheology (flow behavior) of flotation systems, particularly by forming finer and slower bubbles at the sparger or impeller in high-viscosity pulp, resulting in better attachment for fine or hydrophilic particles. Here, bubble formation through an orifice, subsequent growth, and rise in non-Newtonian XG solutions are studied using a computational fluid dynamics (CFD) model. The volume of fluid (VOF) formulation and the continuum surface force (CSF) with the power-law model are used to track and calculate the bubble–liquid interface motion, respectively. The CFD-predicted departure bubble sizes are validated and compared with modified correlations for three different concentrations of XG at three different inlet gas velocities of 0.1–0.3 m s^−1^. The lowest relative error of 0.31–8.93% has been noted against one of the correlation equations. This analysis was extended for the study of contact angle, aspect ratio, and bubble velocity*.* It is found that XG concentrations and inlet gas velocity have a significant impact on bubbles' growth, expansion, elongation, and pinch-off time. The influence of five distinctive dimensionless numbers (*Re*, *Ar*, *Ca*, *We*, and *Mo*) and corresponding forces on bubble formation and growth is also examined. At the end, a comparative study is carried out on the outcomes achieved by using the non-Newtonian power-law model and the Carreau model.

## Abstract 2

Xanthan gum (XG) assists in modifying the rheology (flow behavior) of flotation systems – bubble contact angle, aspect ratio and velocity are strongly influenced by XG concentration and gas velocity.

## Graphical abstract

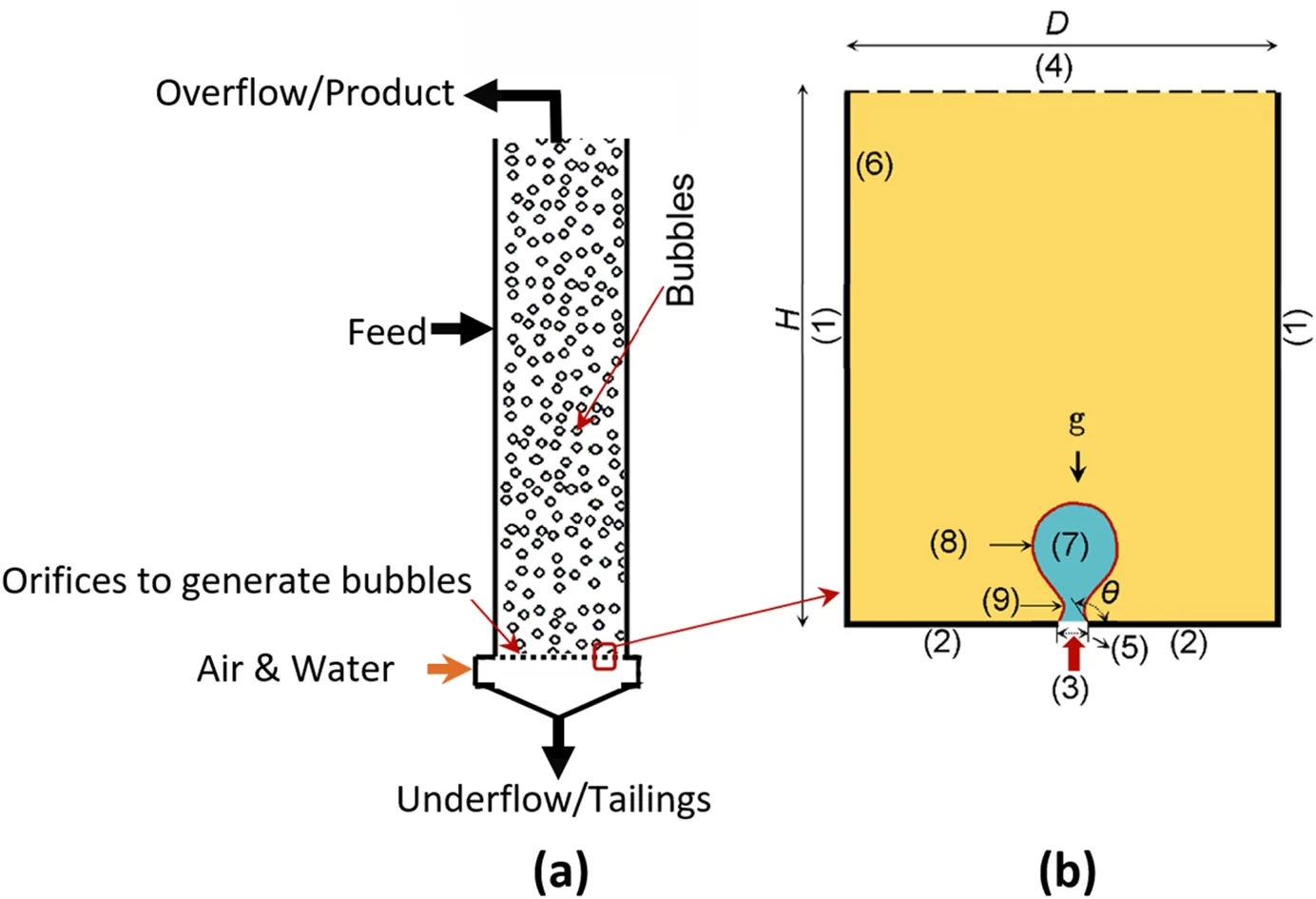

## 1. Introduction

Xanthan gum is a functional, water-soluble extracellular acidic polysaccharide compound secreted by Xanthomonas campestris.^1^ It is used in mineral processing because of its rheology-modifying and selective adsorption properties, which help improve separation efficiency in the flotation of fine sulfides, industrial minerals, and clay-rich ores. It is an effective and selective depressant^2^ for chlorite, enabling the successful separation of arsenopyrite from chlorite, particularly at pulp pH values below 8.^1^ Typically, it modifies bubble formation at spargers or impellers in high-viscosity pulp by producing finer and slower bubbles, which assists with better attachment for fine or hydrophilic particles. Thus, separation efficiency is strongly dependent on bubbles' characteristics such as bubble generation through spargers, which is an arrangement of multiple orifices, bubble size and shape, bubble rise velocity, bubble collision and breakup, *etc.* Researchers have experimentally investigated bubble characteristics in viscoelastic, viscous, and shear-thinning non-Newtonian fluids. A thorough analysis of bubble characteristics results in the efficient design of column flotation and fluidized bed flotation, and other engineering applications. Chen *et al.*^3^ demonstrated that bubble behavior in shear-thinning viscoelastic fluids is complex and deviates significantly from predictions for Newtonian fluids. They also observed that bubbles may rise much more rapidly than expected because of bubble clustering. The findings of the study underscore the importance of considering the non-Newtonian rheology of fluids for applications like flotation, where bubble rise dynamics are crucial.

Peters and Els^4^ experimentally investigated the initial state of the bubble creation. Authors found that the bubble with the mobile surface moves fast. Later, this study was extended to fast and slow bubble rising dynamics. Amirnia *et al.*^5^ studied air bubble rising in aqueous CMC (carboxymethylcellulose) and xanthan gum solution. A high-speed camera was used to determine the terminal velocity of the bubbles. The velocity was found to increase as a function of a power law when small bubbles were focused, and the velocity was constant for large bubbles. Pelletier *et al.*^6^ investigated the bubble dynamics in tap water experimentally. Depending upon the bubble category and radius, the terminal velocity and aspect ratio were obtained. A correlation for restitution when the bubble impacts the wall was developed. The results indicated that the aspect ratio is the primary factor governing bubble–wall impact dynamics. In another study, the bubble terminal rising velocity was analyzed in an aqueous solution of glycerol and water, which was stable.^7^ Using digital imaging, the experiments were performed. The terminal velocity was found to depend highly upon the diameter of the bubble. The terminal velocity, scattering of bubbles, and aspect ratio were reported to change with changing diameter. In glycerin, scattering was not observed, while the terminal velocity increased with diameter. Surface wettability, flow rate, and orifice diameter also play a significant role in bubble formation and detachment.^8^ Moreover, superhydrophobic surfaces tend to generate larger and less frequent bubbles because of strong contact-line pinning, whereas super hydrophilic and untreated aluminum surfaces exhibit comparable bubble behaviors.

CFD has become an efficient tool to study the fundamental insights and to predict the bubble rising characteristics in Newtonian fluids^9,10^ and non-Newtonian fluids.^11,12^ The shear-thinning fluids generally lead to faster bubble rising, and reduce the bubble deformation compared to the Newtonian fluid.^13^ Important parameters like bubbles' terminal velocity, shape, and trajectory are numerically analyzed in a thin bubble column, where experimental observations show that bubbles follow a non-rectilinear path in water but move rectilinearly in non-Newtonian fluids.^14^ Premlata *et al.*^13^ used the volume of fluid (VOF) model to study bubble rising dynamics in non-Newtonian fluid, which was analyzed by adopting the Carreau-Yasuda model. The increase of shear thinning affinity increased the bubble rise velocity and decreased the deformation of the bubble. Eventually, the VOF model was also applied to extract valuable insights into the bubble-assisted transportation of copper matte into the slag phase.^15^ The findings emphasize the critical roles of bubble size, slag properties, and bubble deformation in matte entrainment. Park *et al.*^16^ determined the bubble shape and rising velocity in a liquid pool, such as in radioactive aerosol discharge commonly observed in nuclear power plants. The parameterization of rising velocity was evaluated as a factor of volume equivalent bubble diameter. The characteristics of bubbles were numerically studied using the Carreau model.^17^ The deformation and rise of spheroid bubbles were validated with experimental results. Streamlines, viscosity, and bubble profiles were studied in detail. Xu *et al.*^18^ developed a model for rising bubbles containing α, β, and aspect ratio as parameters to characterize the bubble shape. Numerous influences on bubble deformation were examined, including several forces analysis on the bubble and the fluids' rheological behavior. Rao *et al.*^19^ showed the distinct deformation and breakup behaviors in milli-*versus* micro-channels, highlighting the effect of Eotvos number and nanofluid properties on these phenomena. Compared to the micro-channel, bubble deformation was more prominent in the milli-channel in the presence of shear-thinning liquid. Battistella *et al.*^20^ studied the bubble rising in power-law fluids by applying the Front-Tracking numerical model. Shear-thickening and shear-thinning fluids with a wide range of viscosities were studied for three different bubbles. The viscosity of the fluid affects the shape of bubbles. In shear-thickening fluids, the larger aspherical bubbles tend to become spherical. In shear-thinning fluids, the smaller spherical bubbles lose their sphericity. It was also found that the shear-thinning flow index for different Galilei and Eotvos numbers possesses an influence on bubble aspect ratio, center of gravity, and bubble shape as these evolve over time.^13^

Based on our literature study, previous investigations of non-Newtonian bubble dynamics have largely covered generic shear-thinning or viscoelastic fluids. The present work instead targets Xanthan gum because its polysaccharide structure alters liquid-phase viscosity, interfacial mobility, and the local stress distribution near the gas–liquid interface; these effects determine bubble size, contact angle evolution, and flotation selectivity in mineral suspensions. The novelty of this study therefore lies in (i) coupling bubble-scale CFD predictions directly to XG concentration ranges and flow indices relevant to industrial practice, (ii) benchmarking these predictions against modified Newtonian correlations to quantify the limits of the effective viscosity approach for an actual system, and (iii) directly contrasting the Carreau and power-law descriptions of the same XG solutions rather than treating the choice of rheological model.

## 2. Computational model

### 2.1. Physical model

**Fig. 1:**
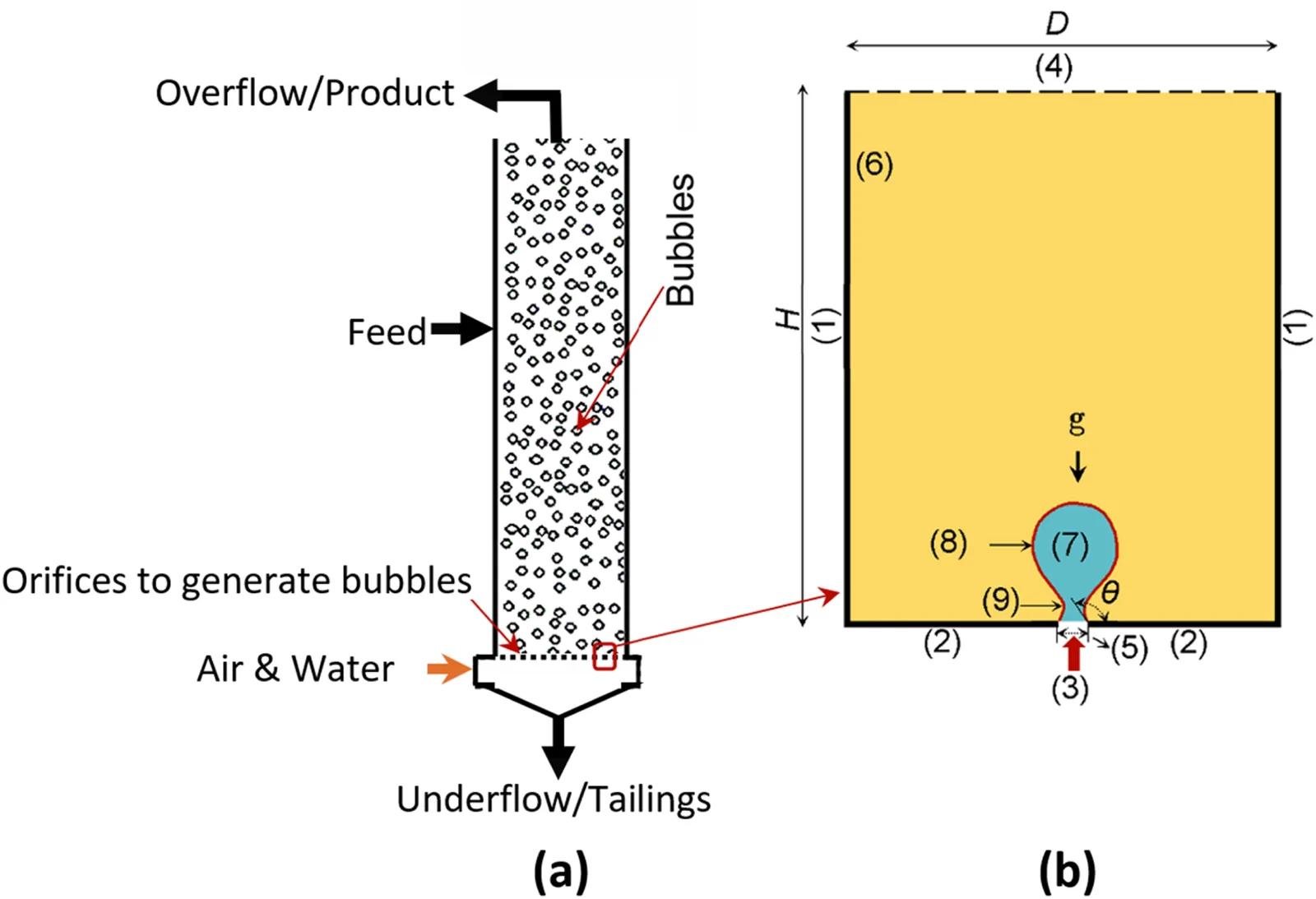
(a) Schematic view of a flotation column and (b) physical model of a bubble formation process – (1) column wall, (2) bottom wall, (3) air inlet, (4) pressure outlet, (5) single orifice (1 mm diameter), (6) liquid or primary phase, (7) air bubble or secondary phase, (8) gas bubble–liquid interface, and (9) bubble neck. *D* and *H* represent the two-dimensional version of the computational domain diameter and height, respectively.

A schematic view of a flotation column and a two-dimensional (2D) domain used for the bubble formation are shown in Fig. 1a and b, respectively. The domain measuring 100 mm in height and 50 mm in width is employed to simulate the formation and flow of a single bubble. The gas inlet is positioned at the bottom wall, utilizing a 1 mm orifice diameter, and is designated as a ‘velocity inlet’ boundary condition (BC). The top wall is defined as the pressure outlet BC, while the side walls are assigned no-slip BCs. Operating pressure is maintained at ambient conditions (101 325 Pa), and gravitational acceleration (*g*) of 9.81 m s^−2^ is applied in the negative *Y* direction. Three different air velocities of 0.1, 0.2, and 0.3 m s^−1^ are selected. These inlet velocities are consistent with values commonly used in industrial spargers; see ref. 21–24, which support the relevance of the selected boundary conditions. Air enters the XG solution *via* the 1 mm orifice, with bubble formation and morphology analyzed numerically. Due to the comparatively small size of the bubble relative to the column width, the influence of the column wall on bubble behavior is negligible.

Here, the 2D approximation is adopted to isolate the coupled effects of XG rheology, gas velocity, and interface deformation at moderate computational cost. This treatment is appropriate for identifying qualitative trends in bubble growth, detachment time, and local viscosity fields under the present isolated bubble conditions. However, the 2D domain suppresses azimuthal instabilities, spiral or wobbling trajectories, and three-dimensional (3D) wake structures; therefore, the predicted results are to be interpreted as representative of the in-plane dynamics rather than as a complete description of 3D bubble motion, and this restriction is regarded as a limitation of the present modeling approach.

### 2.2. Governing equations

#### 2.2.1. Equations of continuity and momentum

The continuity and momentum equations for an incompressible fluid can be written as:^25^1
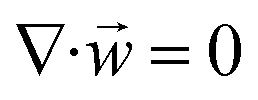
2
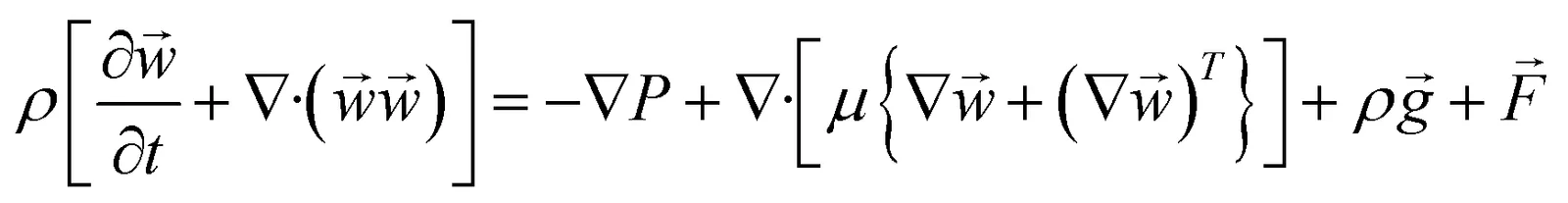
where *P*, *t*, and *g* are the liquid pressure, reference time, and acceleration due to gravity, respectively. Additionally, 
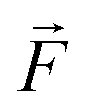
 is the volume force resulting from dynamic stress balance at the gas–liquid interface. Surface tension has an important impact on the interface because the minor curvature of a bubble could generate major additional pressure. The continuum surface force (CSF) is used to calculate the gas–liquid interface motion, which is incorporated as a source term in the momentum equation (eqn (2)) by introducing a body force 
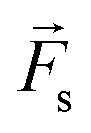
 as described by Brackbill *et al.*^26^ This body force is calculated by the following equation:3
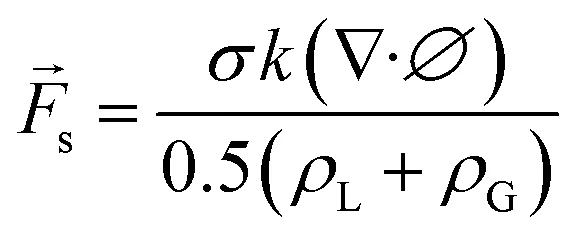
where *σ* is the surface tension. *k* is the surface curvature of the interface, that defined in terms of the divergence of the unit vector, *N̂*, and is described as follows:4
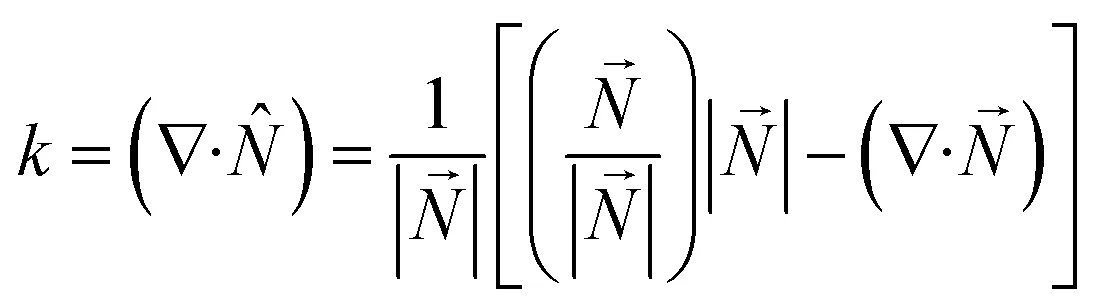


For computation, the XG concentration is selected as the liquid phase, and air is used as the gas phase.

#### 2.2.2. Volume of fluid equations

In the volume of fluid (VOF) formulation, the volume fraction function, *∅*, tracks the gas–liquid interface motion. The value of *∅* is equal to 1, and 0 represents a full liquid and gas phase, respectively, while the value of *∅* between 0 and 1 represents the interface boundary between the gas and the liquid phase. The transport equation is expressed as follows:5
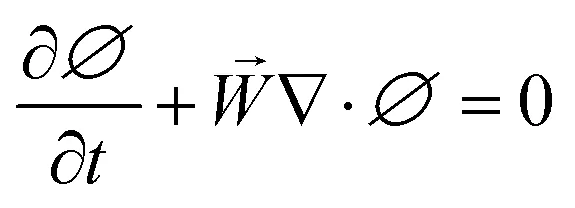
where 
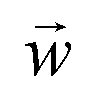
 is the liquid velocity. Here, the piecewise linear interface construction (PLIC) algorithm^27^ is adopted for solving eqn (5) since it has high accuracy in reconstructing the gas–liquid interface. The mixture properties of the interface are described as follows:

6 *ρ* = *ρ*_L_*∅* + *ρ*_G_(1 − *∅*)

7 *µ* = *µ*_L_*∅* + *µ*_G_(1− *∅*)

where *ρ* and *µ* are the density and viscosity of the liquid phase, respectively.

#### 2.2.3. Constitutive equation of liquid

Carreau model: the rheological behavior of the power-law fluid can be described by many constitutive equations. Because the Carreau model can describe the zero-shear-rate and infinite-shear-rate viscosities of the non-Newtonian fluid well, it is extensively applied to describe the rheological behavior of the power-law fluid. Many researchers applied the Carreau model to calculate the apparent viscosity of the liquid phase when they investigated the bubble dynamics in the power-law fluid.^28^ Therefore, the Carreau model is chosen as the constitutive equation of the liquid phase here. It can be seen as follows:8
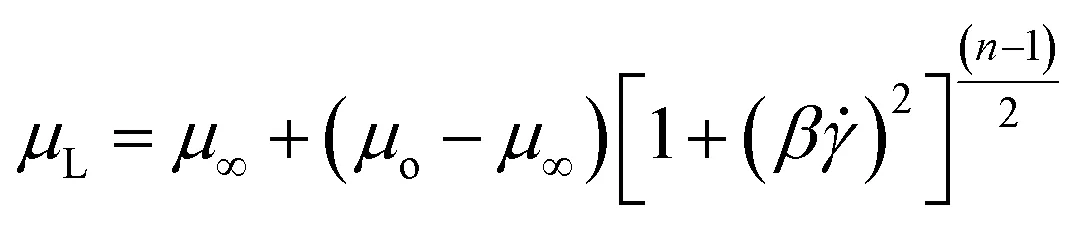
In eqn (8), *β* is the relaxation time constant, and *n* is the rheological index for the non-Newtonian fluid. *µ*_∞_ and *µ*_o_ are the viscosities corresponding to the infinite-shear and zero-shear rate, respectively.

Power-law model: In a non-Newtonian fluid, the shear-thinning effect is presented by the power-law model:^29^

9 *µ*_L_ = *K*(*γ̇*)^*n*−1^

where *K* and *n* are the consistency coefficient and flow index of shear-thinning fluids, respectively. The viscosity *µ* could be obtained from the local shear rate, which is defined as follows:10
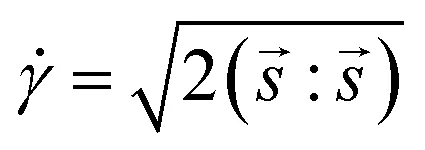


Therefore, the viscosities of non-Newtonian fluids could be obtained by a combination of eqn (3), (9) and (10). Table 1 shows the physical properties of XG concentrations and the purpose of this study.

**Table 1:** Physical properties of the xanthan gum concentration

Solution	Conc. (ppm)	*ρ* (kg m^−3^)	*σ* (mN m^−1^)	*n* (−)	*β* (s)	*K*	*µ*_o_ (Pa s)	*µ*_∞_ (Pa s)	Purpose
XG1	780	998.6	71.9	0.40	4.20	0.13	0.30	0.004	XG effect on different bubble rise characteristics
XG2	1530	998.9	71.5	0.346	5.80	0.30	0.94	0.012	
XG3	2580	999.4	70.5	0.29	16.9	0.64	4.80	0.014	
XG4	1530	998.8	71.8	0.10	5.80	0.19	0.94	0.012	Flow index effect on bubble rise characteristics
XG5	1530	998.8	71.8	0.346	5.80	0.30	0.94	0.012	
XG6	1530	998.8	71.8	0.90	5.80	0.79	0.94	0.012	
XG7	780	998.6	71.9	0.40	0.57	0.42	0.30	0.004	Comparison between *Carreau* model and *power-law* model
XG8	2580	999.4	70.5	0.29	1.43	3.73	4.80	0.0087	

### 2.3. Numerical procedure and mesh dependency study

Ansys-Fluent CFD software,^30^ employing the finite volume method, was utilized to solve the governing equations. To reduce numerical diffusion, the QUICK scheme was implemented for the momentum equation.^29^ Pressure-velocity coupling was accomplished using the pressure implicit with splitting operators (PISO) algorithm, which enables rapid convergence without compromising solution stability or accuracy.^29,31^ The PRESTO! scheme was applied for pressure resolution.^31,32^ The transient model incorporated an explicit scheme, with a time step of 0.0001 s resulting in a Courant number of 0.25. Under-relaxation factors of 0.3 and 0.7 were given for pressure and momentum, respectively, to smooth convergence. A scaled residual of 1 × 10^−6^ was set as the convergence criterion for all governing equations.

Using the same computational geometry (100 mm × 50 mm), Islam *et al.*^33^ performed a mesh-dependence study and validated the predicted single-bubble-rise dynamics and bubble-formation process in water against experimental data from the literature. For the first mesh study of a 4 mm single bubble rise in water, the terminal velocities obtained using the 0.20 mm × 0.20 mm and 0.25 mm × 0.25 mm meshes agreed reasonably closely with experimental data of Raymond and Rosant.^34^ For the second mesh test on bubble formation, the bubble aspect ratio was examined under a superficial gas velocity of 0.2 m s^−1^ through a 1.0 mm orifice diameter. It was noted that these results were taken after the bubble had detached itself from the orifice. Again, the 0.25 mm × 0.25 mm mesh produced smaller differences in the predicted bubble aspect ratio values in comparison to the 0.20 mm × 0.20 mm and 0.30 mm × 0.30 mm sizes of mesh. Therefore, the present study is carried out based on the mesh size of 0.25 mm × 0.25 mm.^33^

## 3. Results and discussion

### 3.1. Validation and comparison with correlation equations

Achieving optimal operating conditions in a fluidized bed flotation separator requires careful consideration of bubble size, as this is a critical parameter. In this system, bubbles are generated at the base *via* a sparger equipped with multiple orifices. Typically, bubble size is assessed immediately following detachment from an orifice. The measurement of gas flow through a known orifice and the timing of bubble detachment allows for estimation of the bubble's departure size.^35^ The equivalent diameter of a bubble at the moment of departure is defined as follows: 
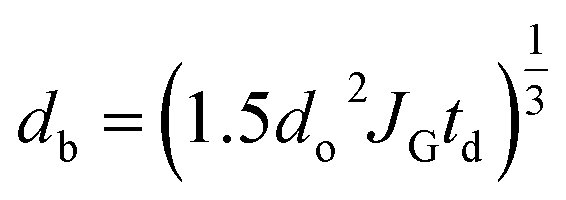
. Here, the departure bubble size under the non-Newtonian XGs fluid is compared with the modified correlation equations (which are available for the Newtonian fluids) by introducing effective viscosity, *µ*_E_. The effective viscosity for the non-Newtonian fluid is written as follows:^36^11
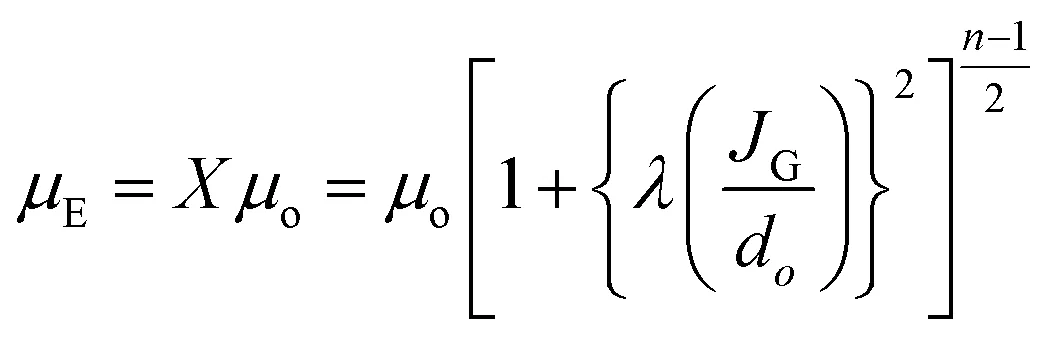
In eqn (11), the bubble formation in Newtonian flow can be described by the value of *X* = 1, so the effective viscosity of the liquid is equal to the liquid viscosity, *µ*_L_. Hence, the empirical correlations developed by ref. 37–39 respectively, are modified and written as –12
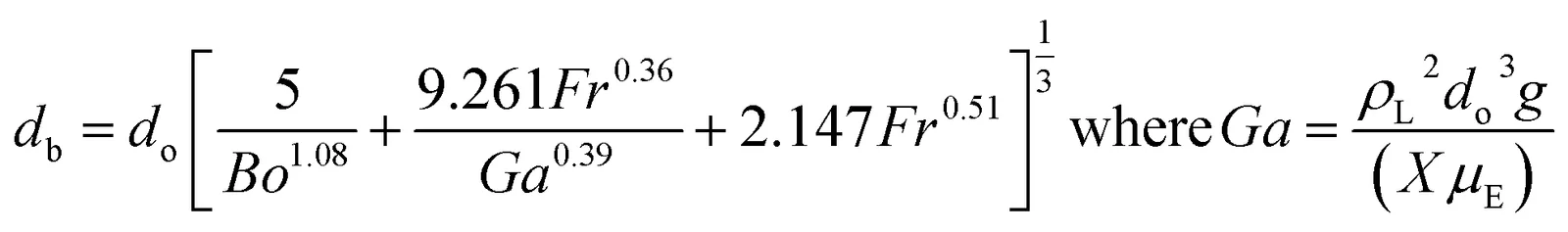
13
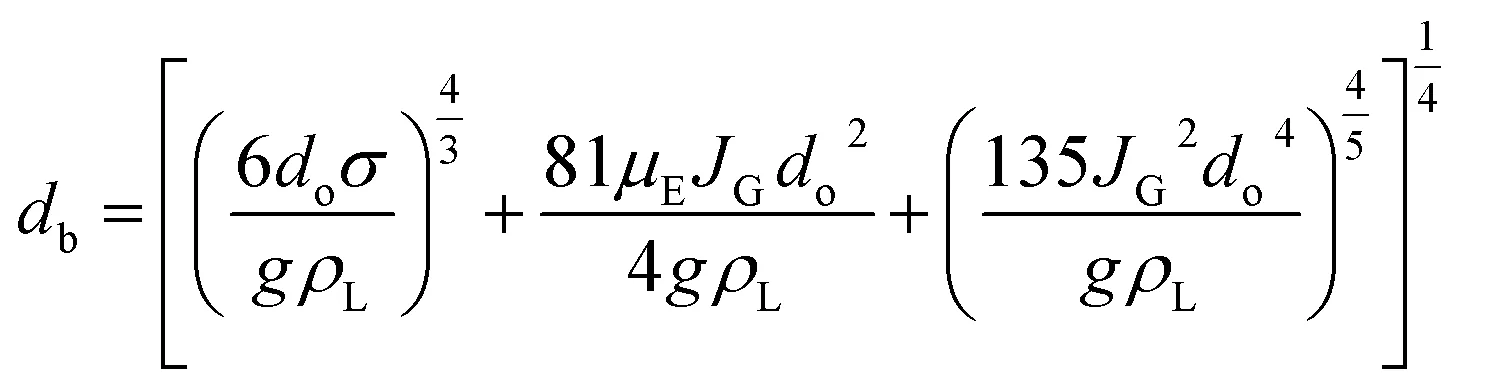
14
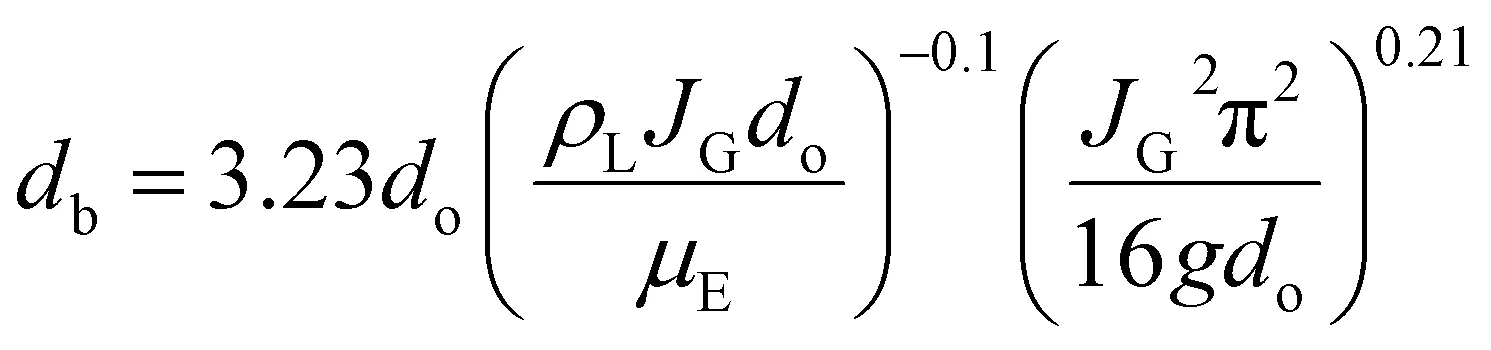


**Fig. 2:**
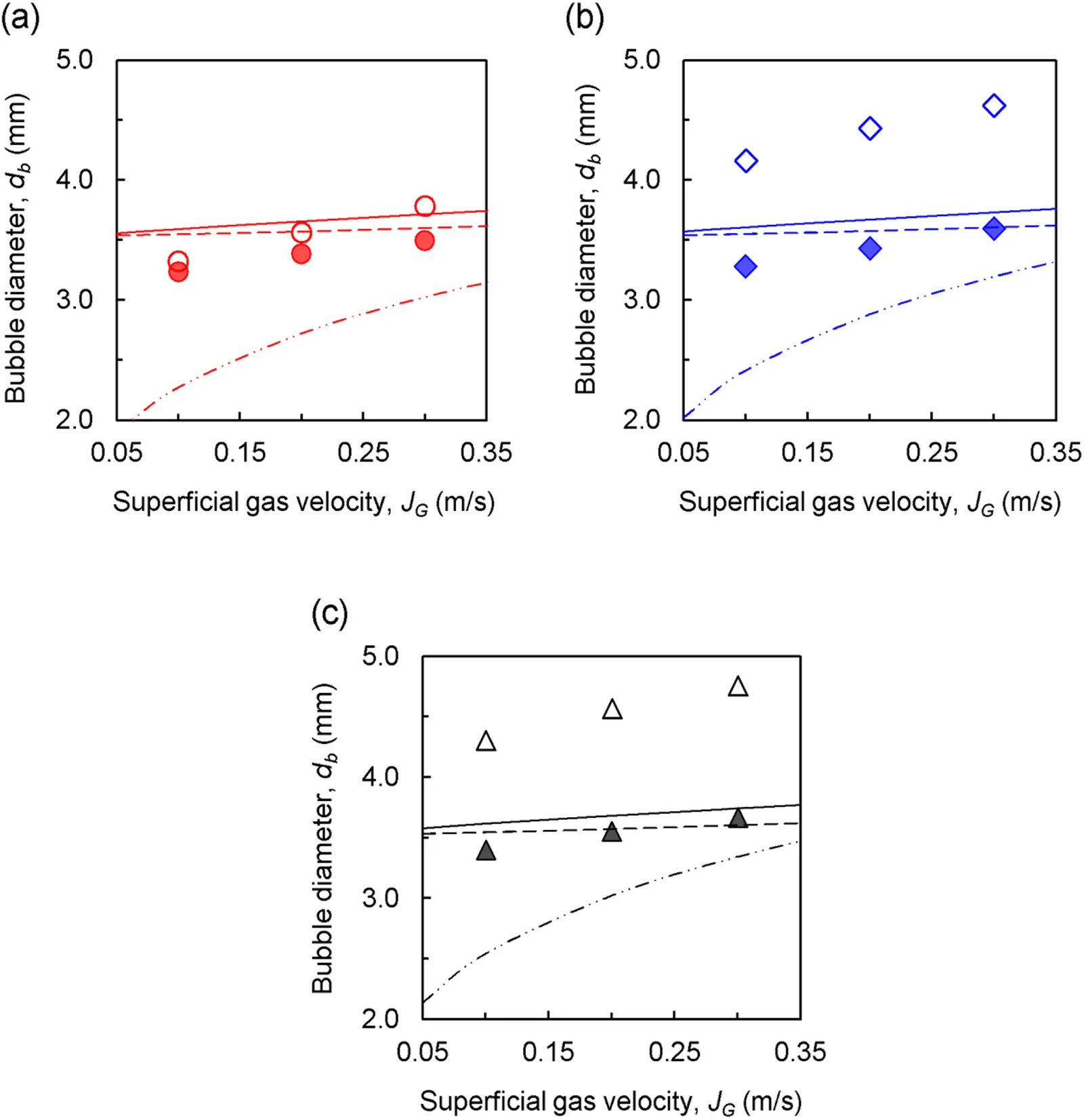
Comparison between equivalent bubble diameter and correlation results of (a) 780 ppm, XG1, (b) 1530 ppm, XG2, and (c) 2580 ppm, XG3. Filled and unfilled symbols represent the bubble diameter based on the departure time and the pinch-off of the bubble, respectively. Solid, dashed, and double dot with dashed lines define the calculated results using eqn (12)–(14), respectively.

Fig. 2 illustrates the correlation between bubble size or diameter at departure and the superficial gas velocity. The results indicate that as gas velocity increases, so does the bubble diameter. The CFD results closely match the predictions of the correlations reported in ref. 37 and 38. Conversely, the correlation reported in ref. 39 shows considerable deviation, attributed to its exclusion of surface tension forces. Table 2 shows the relative errors between CFD results and predicted results using three correlations for different XG concentrations. The ranges of error using eqn (12)–(14) are 10.08–1.95%, 8.93–0.31%, and 42.02–9.72%, respectively. Moreover, Fig. 2 also shows superficial gas velocity *versus* bubble diameters based on the departure time (filled symbols) and the pinch-off of the bubble (which is calculated after pinch-off from the orifice and denoted by unfilled symbols), respectively. For 780 ppm XG, the departure diameters are similar to the bubble pinch-off diameters; however, for 1530 and 2580 ppm XG, the pinch-off diameters were up to 52% greater than the departure diameters. These results highlight the significant influence of non-Newtonian fluid concentration on bubble size.

**Table 2:** Relative errors between CFD and correlations for different XG

	Error in % with eqn (12)			Error in % with eqn (13)			Error in % with eqn (14)		
	*J*_G_ *=* 0.1 m s^−1^	0.2 m s^−1^	0.3 m s^−1^	0.1 m s^−1^	0.2 m s^−1^	0.3 m s^−1^	0.1 m s^−1^	0.2 m s^−1^	0.3 m s^−1^
XG1	10.08	7.45	5.98	8.93	5.25	3.02	42.02	24.21	15.37
XG2	9.06	6.60	3.68	7.58	4.07	0.31	35.77	18.97	12.46
XG3	6.00	3.46	1.95	4.07	0.46	1.83	33.64	17.58	9.72

Besides, the substitution of the effective viscosity into Newtonian bubble size correlations is used here as an approximation for shear-thinning liquids. Its validity is expected to be strongest when the dominant shear rates near the orifice are represented reasonably by the effective viscosity and when elastic effects remain secondary. The approach partially captures the spatial variation of viscosity around the growing neck, but cannot replace direct experimental validation in XG solutions; therefore, the correlation errors reported below are interpreted as a consistency check on the CFD trend rather than a complete proof of predictive accuracy. Future work incorporating direct experimental bubble size data for XG would allow this approximation to be tested independently.

It is noted that with the same computational geometry (100 mm × 50 mm) and mesh size (0.25 mm × 0.25 mm) conditions, Tariqul *et al.*^33^ have done the mesh dependency study and the experimental validation with the literature data for a single bubble rising velocity and bubble formation process in water. The maximum difference was less than 3.2% in comparison between the 4 mm and 5 mm bubbles' terminal velocity and the experimental data.^34^ The shape of the bubble during formation stages through a 1.0 mm orifice at a superficial gas velocity of 0.2 m s^−1^ agreed excellently with the experimental data of Davidson *et al.*^40^

This study explores the bubble formation phenomenon in non-Newtonian XG concentrations based on the same computational geometry and mesh size used by Islam *et al.*^33^ As sufficient experimental data on bubble formation in non-Newtonian XG solutions are unavailable in the literature, the present CFD results are assessed against modified correlation equations extended to non-Newtonian fluids.

### 3.2. Dominant forces on bubble growth

Bubble formation, growth, pinch-off, and rising are balanced by different forces. Here, we analyzed the force that dominates the bubble growth in terms of different dimensionless numbers. In general, bubble formation and growth behavior are the result of the interaction between various forces such as inertial, viscous drag, gravitational, surface tension, and buoyancy. In the absence of wall effects on bubble formation and growth, it is characterized by the dimensionless numbers, such as the Reynolds number (measurement of the inertial force of the flow to the viscosity force), 
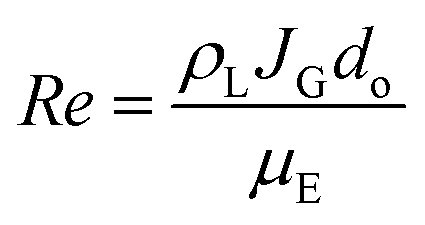
; Archimedes number (buoyancy to viscosity force), 
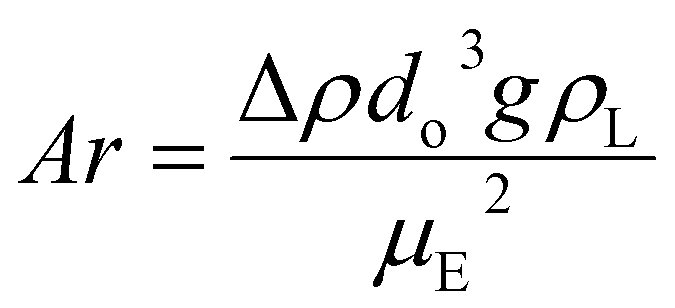
; Capillary number (viscous drag to surface tension force), 
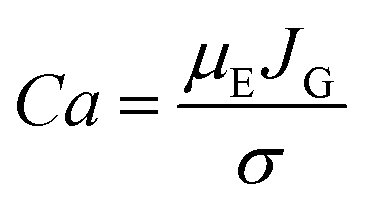
; Weber number (inertial to surface tension force) 
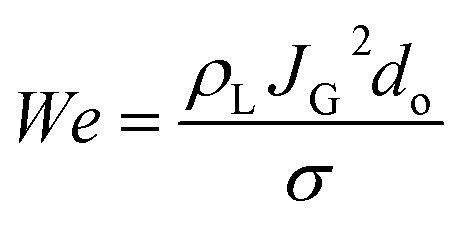
; and Morton number (physical properties of the liquid phase), 
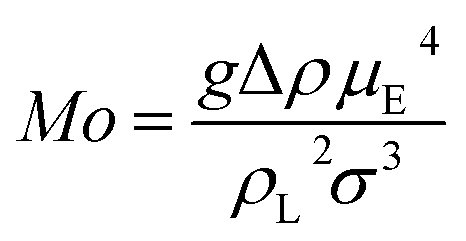
^41,42^ To understand the forces influencing bubble formation and growth in XG, dimensionless numbers at the orifice were derived using effective viscosity, and their importance within the range of parameters considered here was discussed.

Tables 3 and 4 show the numerical values of these dimensionless numbers and the dominant force on bubble formation and growth stages, respectively. As XG concentration increases (Case XG1 to XG3), both Reynolds (*Re*) and Archimedes (*Ar*) numbers decrease greatly due to higher effective viscosity. This signifies that the viscous fluid medium increasingly hinders bubble movement, with the lowest values observed in XG3 at the highest XG concentration. Increasing gas velocity leads to higher *Re* across all cases, indicating the upward dominance of inertial over viscous forces. For XG1 at *J*_G_ = 0.2 and 0.3 m s^−1^ and for XG4 across the selected velocities, the *Ar* number exceeds 100, indicating buoyancy-dominated behavior under those specific conditions. On the contrary, the *Ca*, *We*, and *Mo* numbers increase notably at higher XG concentration. Similarly, an increase is observed in the *Ca* and *We*, while a noticeable decline in the *Mo* is observed at higher velocity. In the case of *Ca*, the trend shows the impact of surface tension force on the bubble dynamics; a similar phenomenon is also noticed in the *We* at 0.1 and 0.2 m s^−1^ inlet air velocities. However, at 0.3 m s^−1^, the inertia force exceeds the effect of the surface tension force.

**Table 3:** Influence of dimensionless numbers on bubble formation and growth

	*J*_G_ = 0.1 m s^−1^					*J*_G_ = 0.2 m s^−1^					*J*_G_ = 0.3 m s^−1^				
	*Re*	*Ar*	*Ca*	*We*	*Mo*	*Re*	*Ar*	*Ca*	*We*	*Mo*	*Re*	*Ar*	*Ca*	*We*	*Mo*
XG1	8.11	64.59	0.017	0.139	6.1 × 10^−7^	24.60	148.39	0.023	0.556	1.1 × 10^−7^	47.06	241.38	0.027	1.250	4.3 × 10^−8^
XG2	3.84	14.45	0.036	0.140	1.2 × 10^−5^	12.08	35.77	0.046	0.559	2.0 × 10^−6^	23.62	60.79	0.053	1.257	7.0 × 10^−7^
XG3	1.49	2.19	0.095	0.142	5.6 × 10^−4^	4.89	5.86	0.116	0.567	7.8 × 10^−5^	9.78	10.42	0.130	1.276	2.5 × 10^−5^
XG4	14.79	214.52	0.009	0.139	5.5 × 10^−8^	55.19	746.98	0.010	0.556	4.5 × 10^−9^	119.24	1549.8	0.010	1.252	1.1 × 10^−9^
XG5	3.84	14.44	0.036	0.139	1.2 × 10^−5^	12.08	35.76	0.046	0.556	2.0 × 10^−6^	23.61	60.78	0.053	1.252	6.9 × 10^−7^
XG6	0.18	0.03	0.757	0.139	2.3	0.39	0.04	1.412	0.556	1.7	0.62	0.04	2.033	1.252	1.5
XG7	4.46	19.50	0.031	0.139	6.6 × 10^−6^	13.52	44.81	0.041	0.556	1.3 × 10^−6^	25.86	72.88	0.048	1.250	4.8 × 10^−7^
XG8	0.62	0.38	0.228	0.142	1.9 × 10^−2^	2.03	1.01	0.279	0.567	2.6 × 10^−3^	4.07	1.80	0.314	1.276	8.3 × 10^−4^

**Table 4:** Dominant force on bubble formation and growth^a^

	*J*_G_ = 0.1 m s^−1^				*J*_G_ = 0.2 m s^−1^				*J*_G_ = 0.3 m s^−1^			
	*Re*	*Ar*	*Ca*	*We*	*Re*	*Ar*	*Ca*	*We*	*Re*	*Ar*	*Ca*	*We*
XG1	*F*_in_	*F*_vis_	*F*_suf_	*F*_suf_	*F*_in_	***F***_**b**_	*F*_suf_	*F*_suf_	*F*_in_	***F***_**b**_	*F*_suf_	***F***_**in**_
XG2	*F*_in_	*F*_vis_	*F*_suf_	*F*_suf_	*F*_in_	*F*_vis_	*F*_suf_	*F*_suf_	*F*_in_	*F*_vis_	*F*_suf_	***F***_**in**_
XG3	*F*_in_	*F*_vis_	*F*_suf_	*F*_suf_	*F*_in_	*F*_vis_	*F*_suf_	*F*_suf_	*F*_in_	*F*_vis_	*F*_suf_	***F***_**in**_
XG4	*F*_in_	***F***_**b**_	*F*_suf_	*F*_suf_	*F*_in_	***F***_**b**_	*F*_suf_	*F*_suf_	*F*_in_	***F***_**b**_	*F*_suf_	***F***_**in**_
XG5	*F*_in_	*F*_vis_	*F*_suf_	*F*_suf_	*F*_in_	*F*_vis_	*F*_suf_	*F*_suf_	*F*_in_	*F*_vis_	*F*_suf_	***F***_**in**_
XG6	***F***_**vis**_	*F*_vis_	*F*_suf_	*F*_suf_	***F***_**vis**_	*F*_vis_	***F***_**d**_	*F*_suf_	***F***_**vis**_	*F*_vis_	***F***_**d**_	***F***_**in**_
XG7	*F*_in_	*F*_vis_	*F*_suf_	*F*_suf_	*F*_in_	*F*_vis_	*F*_suf_	*F*_suf_	*F*_in_	*F*_vis_	*F*_suf_	***F***_**in**_
XG8	***F***_**vis**_	*F*_vis_	*F*_suf_	*F*_suf_	*F*_in_	*F*_vis_	*F*_suf_	*F*_suf_	*F*_in_	*F*_vis_	*F*_suf_	***F***_**in**_

a Inertia, viscous, surface tension, buoyancy and drag forces are denoted by *F*_in_, *F*_vis_, *F*_suf_, *F*_b_ and *F*_d_, respectively.

Variation of the flow index significantly influences the dimensionless numbers on bubble formation and growth. At a uniform 1530 ppm concentration, XG4, XG5, and XG6, with the flow index *n* = 0.1, 0.346, and 0.9, respectively, represent their influence on different dimensionless numbers. For the mentioned three velocities, the lowest *n* (XG4) achieves the highest values for *Re* and *Ar*, which indicates the prominent impact of the inertia force and buoyancy force, respectively. As the flow index increases, Re number decreases significantly which implies the impact of viscous force over inertia force. A similar trend is observed in the case of the *Ar* number, where at the medium and high flow index, the viscous force is dominant over the buoyancy force. In contrast, the capillary number increases with the increase of *n* and inlet jet velocity, but at 0.1 m s^−1^ in all cases, the surface tension force remains dominant across all cases. Further consideration of the *Ca* shows that, at *J*_G_ = 0.2 and 0.3 m s^−1^, drag forces surpass surface tension at *n* = 0.9. The *We* numbers give minimal sensitivity to changes in *n*, instead varying only with XG concentrations and *J*_G_. At *J*_G_ = 0.3 m s^−1^, inertia dominates surface tension. An increase is found in the *Mo* as *n* increases, exceeding the value of 1 for *n* = 0.9 (XG6) at all three *J*_G_ cases.

Two separate XG concentrations, XG7 (780 ppm) and XG8 (2580 ppm), were evaluated using the Carreau and power-law models, respectively, to assess their effects on the dimensionless numbers. The Carreau model demonstrated that the *Re*, *Ar*, *Ca*, and *We* numbers all increase as *J*_G_ increases. For each respective number, inertia, viscous, and surface tension forces predominated, apart from for the *We* number at *J*_G_ = 0.3 m s^−1^, where inertia surpassed surface tension as the dominant force. In contrast, the power-law model indicated that viscous forces had greater influence over XG8 at *J*_G_ = 0.1 m s^−1^; however, as *J*_G_ increased, inertia became the prevailing force. The power-law model also demonstrated similar dominant force tendencies with increasing *J*_G_. Across both models, the *Mo* number steadily dropped as *J*_G_ increased.

### 3.3. Effect of air velocity, *J*_G_

**Fig. 3:**
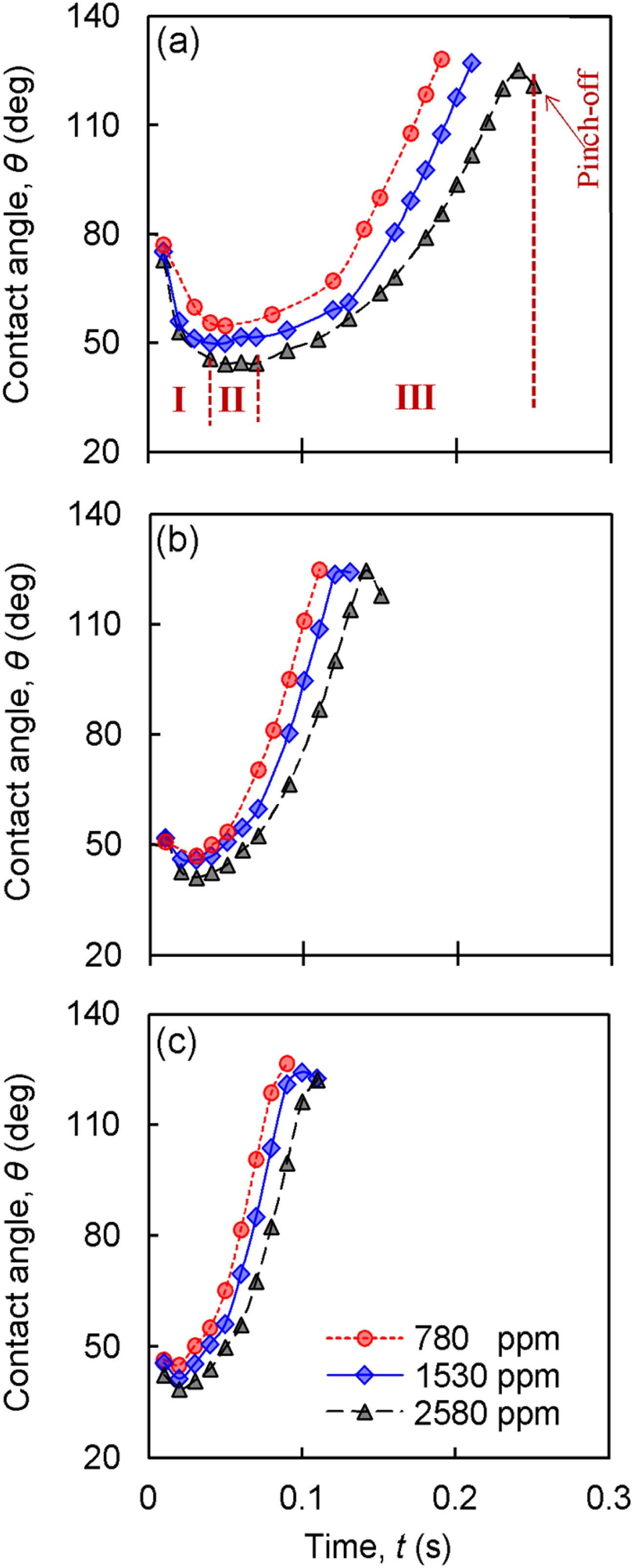
Bubble formation history (*θ*) in different XG at (a) *J*_G_ = 0.1 m s^−1^, (b) *J*_G_ = 0.2 m s^−1^, and (c) *J*_G_ = 0.3 m s^−1^. In top figure, history of bubble growth stage, expansion and elongation stages are defined by I, II, and III, respectively.

Fig. 3 shows the variation in contact angle (*θ*) throughout the bubble formation process for three different XG concentrations at three *J*_G_ velocities. The *θ* between the orifice base and the bubble surface undergoes rapid changes, which can be categorized into three stages, namely bubble growth, expansion, and elongation stages. As shown in Fig. 3a, at *J*_G_ = 0.1 m s^−1^ across all XG, the *θ* decreases rapidly from approximately 80° due to initial bubble growth. Buoyancy force drastically contributes to elevating the bubble, further influencing the decline in *θ*. Upon reaching its minimum value, the *θ* remains steady temporarily while the bubble reaches its maximum potential volume. In the final stage, increasing buoyancy on the bubble's upper surface governs the bubble upward, resulting in thinning near the orifice and generating a bottleneck shape. During this stage, the *θ* gradually increases, eventually forming an obtuse angle as the bubble detaches and rises. The *θ* profile lowers with higher XG, and pinch-off time extends correspondingly. Fig. 3b and c show that increased *J*_G_ results in similar trends, though the periods of the growth, expansion, and elongation stages decrease distinctly. After the initial sharp decay during the growth, bubbles rise rapidly, forming a larger contact angle at higher *J*_G_ before detaching from the orifice. Subsequently, the expansion stage duration becomes short at higher *J*_G_ that it is difficult to illustrate in the figure. Higher *J*_G_ also causes a notable drop in pinch-off time. For XG = 2580 ppm, pinch-off occurs at around 0.1 s, 0.15 s, and 0.25 s at *J*_G_ = 0.3 m s^−1^, 0.2 m s^−1^, and 0.1 m s^−1^, respectively. Additionally, Fig. 3 indicates that increasing both *J*_G_ and XG concentration leads to decreased *θ* during both bubble growth and expansion stages.

**Fig. 4:**
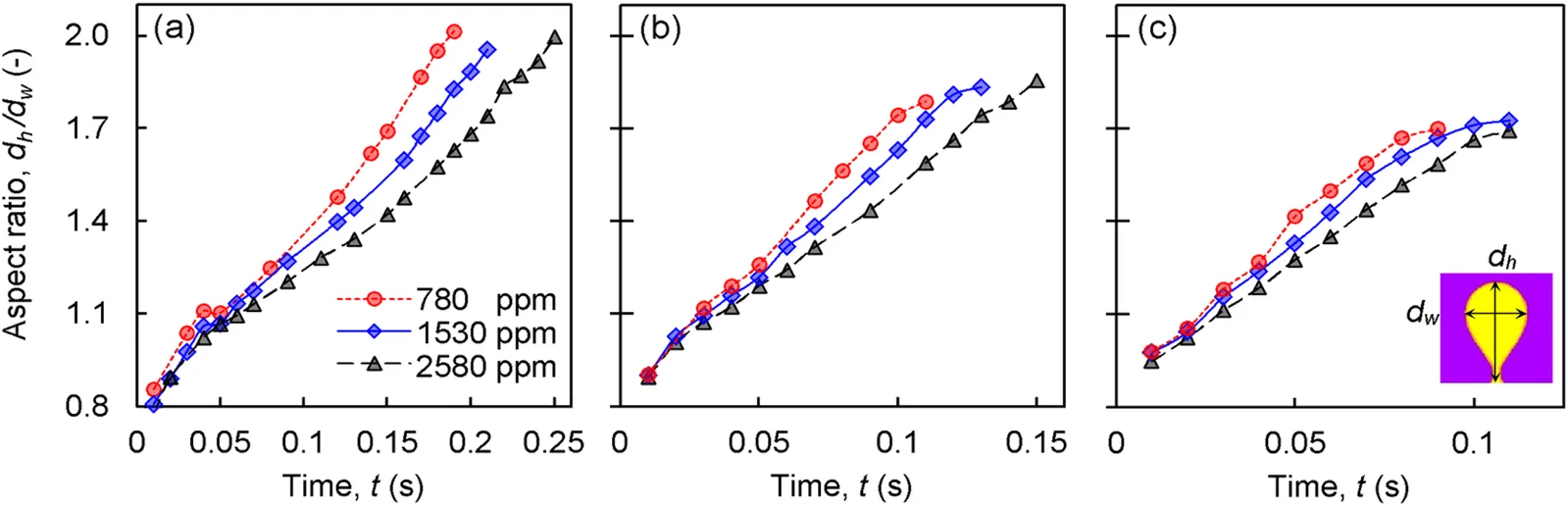
Bubble aspect ratio *versus* time in different concentrations of XG at (a) *J*_G_ = 0.1 m s^−1^, (b) *J*_G_ = 0.2 m s^−1^, and (c) *J*_G_ = 0.3 m s^−1^.

Changes in bubble shape in three different XG concentrations are depicted in Fig. 4 through the variation in aspect ratio. For all three XG concentrations, the aspect ratio increases over time, which means the bubble elongates as it rises. In the 780 ppm XG solution (Fig. 4a), the aspect ratio increases at a faster rate, and with the increase in XG concentration, the bubbles' deformation takes place slowly. Despite this, the overall upward trend in aspect ratio remains consistent across different concentrations. *J*_G_ is observed to have key effects on bubble aspect ratios: higher *J*_G_ results in less elongation of bubbles. Fig. 4a–4c show that the maximum aspect ratio in the 2580 ppm XG is roughly 2.0, 1.85, and 1.7 for *J*_G_ of 0.1 m s^−1^, 0.2 m s^−1^, and 0.3 m s^−1^, respectively. Additionally, the time required for shape deformation decreases significantly, with peak aspect ratios occurring at 0.25 s, 0.15 s, and 0.12 s for the respective velocities.

**Fig. 5:**
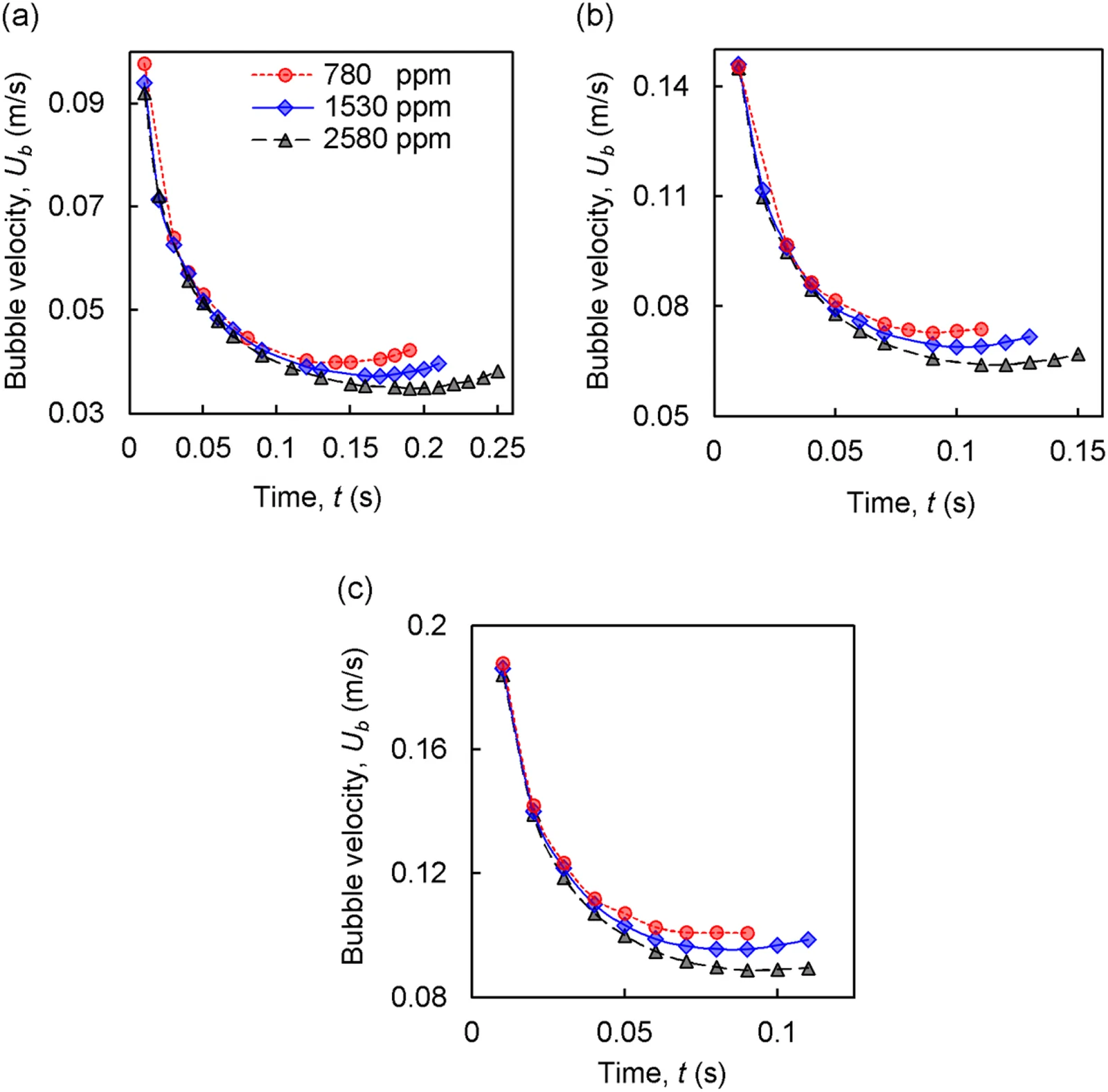
Bubble velocity *versus* time in different concentrations of XG at (a) *J*_G_ = 0.1 m s^−1^, (b) *J*_G_ = 0.2 m s^−1^, and (c) *J*_G_ = 0.3 m s^−1^.

Fig. 5a–c show the variation in bubble velocities for three XG concentrations at *J*_G_ of 0.1–0.3 m s^−1^, respectively. An increase in *J*_G_ results in a corresponding rise in the initial bubble velocity for all cases. The XG concentration significantly affects bubble velocity within the fluid column; specifically, higher XG concentration leads to a reduction in terminal velocity, with the lowest final velocity observed in the highest XG concentration. Particularly, bubbles achieve a certain velocity more rapidly in solutions with higher XG. For each *J*_G_, the bubble velocity profile follows a consistent pattern, characterized by a sharp initial decline followed by a gradual decrease throughout the remaining period.

**Fig. 6:**
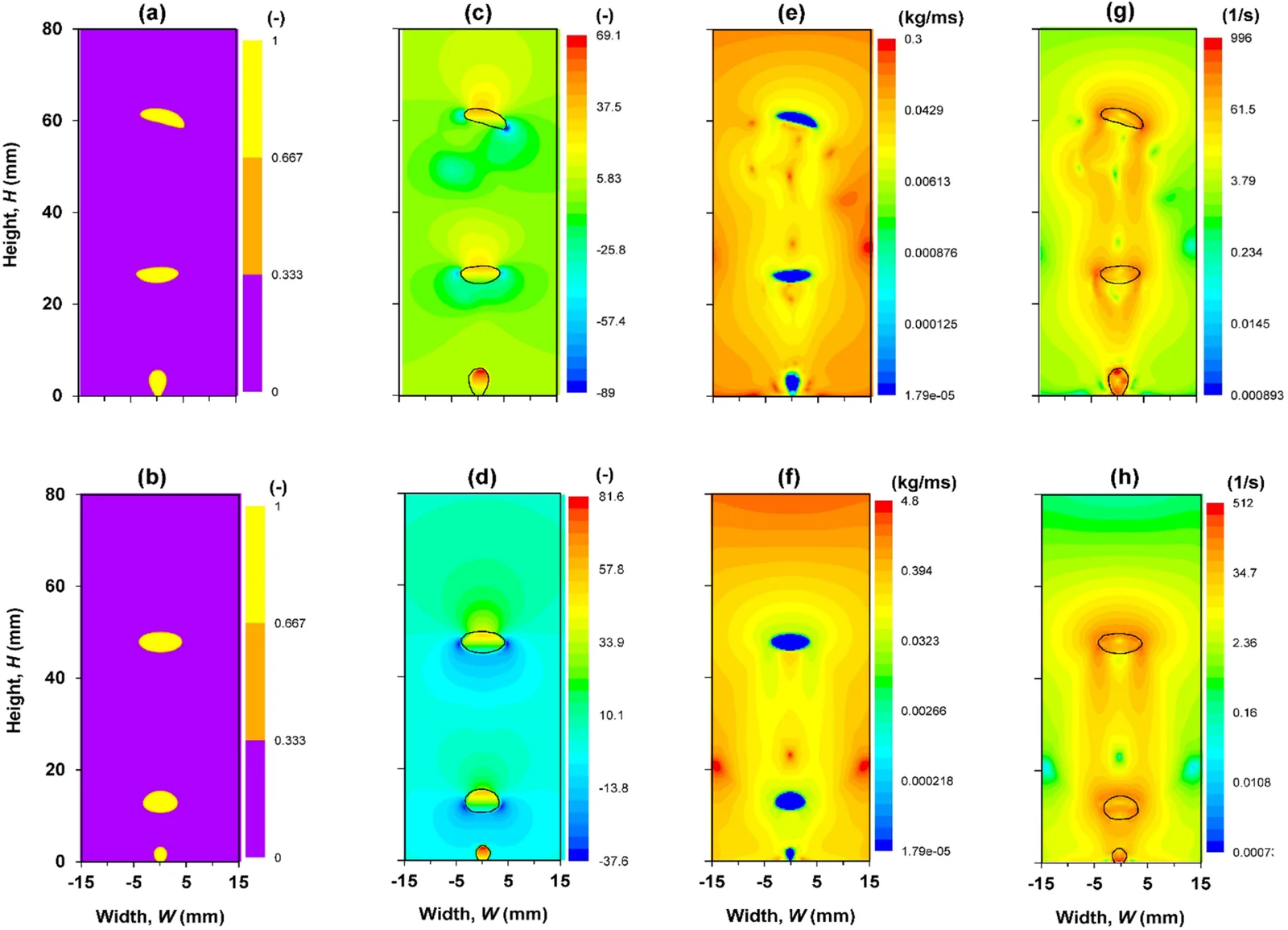
(a & b) Bubble shape, (c & d) pressure coefficient, (e & f) molecular viscosity, and (g & h) shear rate around the bubble for *J*_G_ = 0.1 m s^−1^ (top row) XG1: *Re* = 8.11, *Ar* = 64.59, *Bo* = 0.136, *We* = 0.139, *Mo* = 6.1 × 10^−7^ and (bottom row) XG3: *Re* = 1.49, *Ar* = 2.19, *Bo* = 0.139, *We* = 0.142, *Mo* = 5.6 × 10^−4^.

The effect of the different concentrations of XG on the bubble formations is illustrated in Fig. 6, at *J*_G_ = 0.1 m s^−1^. The change in XG concentration causes significant changes in the bubble morphology, as Fig. 6a and b indicate. It is observed from Fig. 6a, at a lower XG concentration (780 ppm), the leading bubble reaches a height of 60 mm due to buoyancy at a specific time, with an oblate ellipsoidal shape in the upper surface and maintaining a predominant horizontal lower surface. Meanwhile, the second bubble with the shape of an oblate ellipsoid reaches approximately 30 mm, and the third bubble is at the elongation stage. On the contrary, as depicted in Fig. 6b, due to the higher viscosity of the XG, the leading bubble at the same time reaches nearly 50 mm, with its elongated major axis remaining horizontal. Besides, the second bubble has a more rounded shape, whereas the third bubble is at the growth stage.

Fig. 6c and d show the influence of the pressure coefficient on the bubbles at different heights. In lower and higher-concentration XG solutions, an analogous scenario is observed: a higher-pressure coefficient is observed on the upper side of the inside of the bubbles, and a comparatively lower pressure coefficient occurs on the lower interior of the bubbles. This pressure distribution causes the bubbles to acquire an upward motion that has to counter the force of gravity as well as the viscous force of the XG solutions, leading to the high pressure in the upper areas. Outside of the bubble's surface, a substantially lower pressure coefficient acts than inside the bubble. With an increase in the XG concentration, the pressure coefficient also increases. In both cases, the highest-pressure coefficient acts on the bubble, which is in the growth stage, and negative pressure acts on the left and right edges of the leading and second bubbles.

Fig. 6e and f visualize the molecular viscosity field around the bubbles in two different XG solutions, demonstrating obvious shear-thinning characteristics. In both cases, the local viscosity around the moving bubbles drops, which facilitates the ascent of the bubbles and stabilizes their shape and motion. In the higher-concentrated solution, the viscosity around the bubble is higher than in the lower-concentrated XG solution. Comparatively higher viscosity is observed in the middle region of the bubble underneath in all cases.

To understand bubble rise hydrodynamics, it is important to analyze the shear rate distribution around the bubble. Fig. 6g and h, respectively, illustrate this property for lower and higher XG concentrations, respectively, at a velocity of 0.1 m s^−1^ and with the increase of concentration, a noticeable drop in net shear rate is observed. Both figures indicate that the shear rate around the bubble is substantially higher than in the closest regions, with the upper side of each bubble exhibiting a consistently similar distribution in both cases. The shear rate beneath the bubble varies significantly depending on the XG concentration. As depicted in Fig. 6h, an extended linear tail with elevated shear rates forms near the left and right sides below the bubble, while comparatively lower shear rates are observed near the bubble's core. In lower XG, the shear rate distribution around the leading bubble adheres to a similar trend; however, the areas of increased shear rate on both sides develop a curved profile, as opposed to the straight tails found in higher XG.

### 3.4. Effect of liquid flow index, *n*

**Fig. 7:**
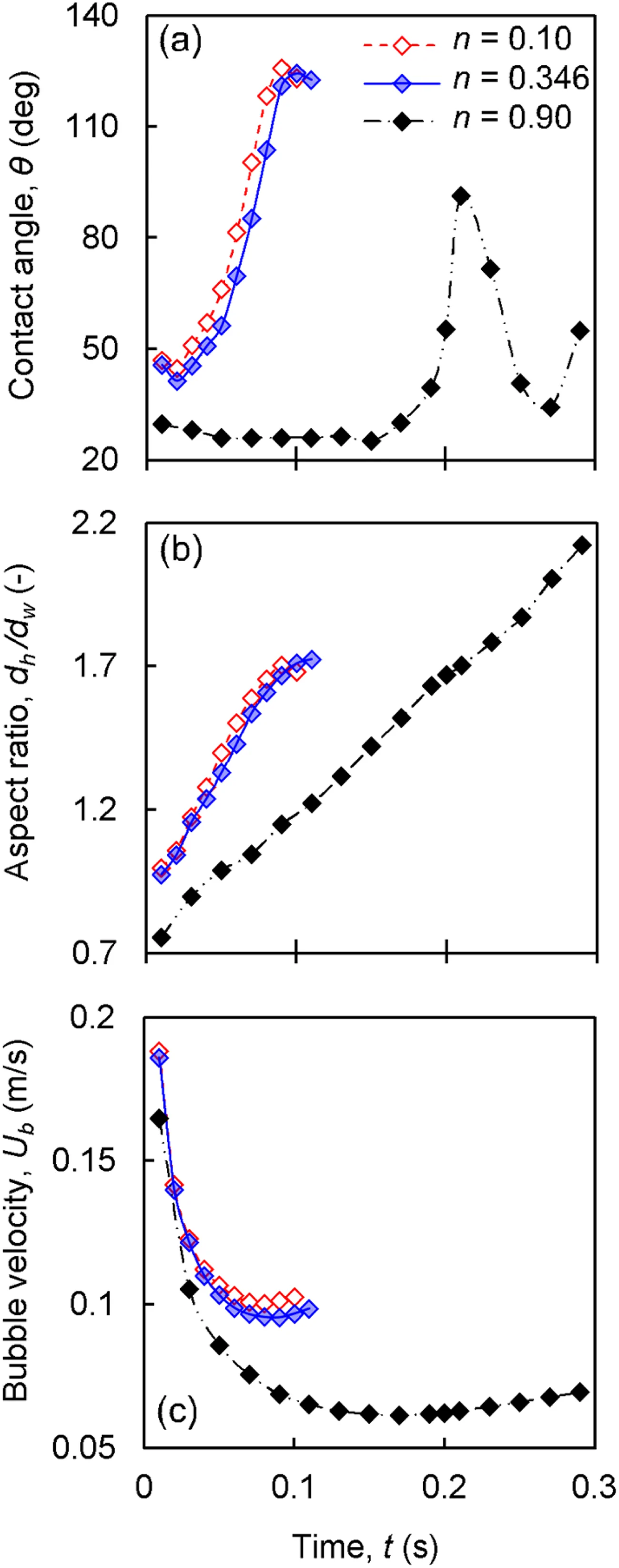
Influence of flow index (*n*) of Carreau model on bubble (a) contact angle, (b) aspect ratio, and (c) velocity for the concentration of 1530 ppm at *J*_G_ = 0.3 m s^−1^.

According to the Carreau model, the impact of the liquid flow index (*n*) is shown in Fig. 7. As shown in Fig. 7a, the variation in *θ* with respect to changes in *n* reveals that both low (*n =* 0.10) and medium (*n =* 0.346) flow indices exhibit similar patterns: pinch-off occurs around 0.1 s, and elongation begins immediately following the growth stage. In contrast, a higher *n =* 0.90, which is nearly 3-fold greater than the medium value, demonstrates a distinct trend characterized by a maintained reduction in *θ* and an increased pinch-off duration. The expansion phase lasts an extended period following the growth phase, and at a period of about 0.2 seconds, the steep rise in contact angle leads to the beginning of the elongation phase. Besides, towards pinch-off (around 0.3 seconds), the contact angle variability is detected at the elevated flow index.

Morphology of bubbles is also influenced by the flow index. According to Fig. 7b, the profiles of the aspect ratio of the low and medium flow indices are very similar: at the beginning of the time, the aspect ratio is 1, which corresponds to a spherical bubble, and it increases with time as the bubble changes its shape to an oval or ellipsoidal one. By contrast, with the high flow index (*n* = 0.90), the initial aspect ratio is less than 1, so the bubble is horizontally elongated or oblate, and becomes more vertically elongated as the aspect ratio increases to more than 2 at detachment.

The flow index also affects the bubble velocity. With increasing flow indices, the velocity of the bubbles is significantly reduced, but the general velocity curves are the same at all indices and are shown in Fig. 7c. Both low and medium flow indices have a starting bubble velocity of about 0.19 m s^−1^, after which it slows to below 0.1 m s^−1^ abruptly, and then remains steady towards pinch-off. On the contrary, when the flow index is large (*n* = 0.90), the initial velocity is approximately 0.17 m s^−1^ and soon drops to less than 0.10 m s^−1^ because of slowing down. This is followed by a slowing down of speed to 0.06 m s^−1^ in 0.2 seconds and then a slight rise towards detachment.

**Fig. 8:**
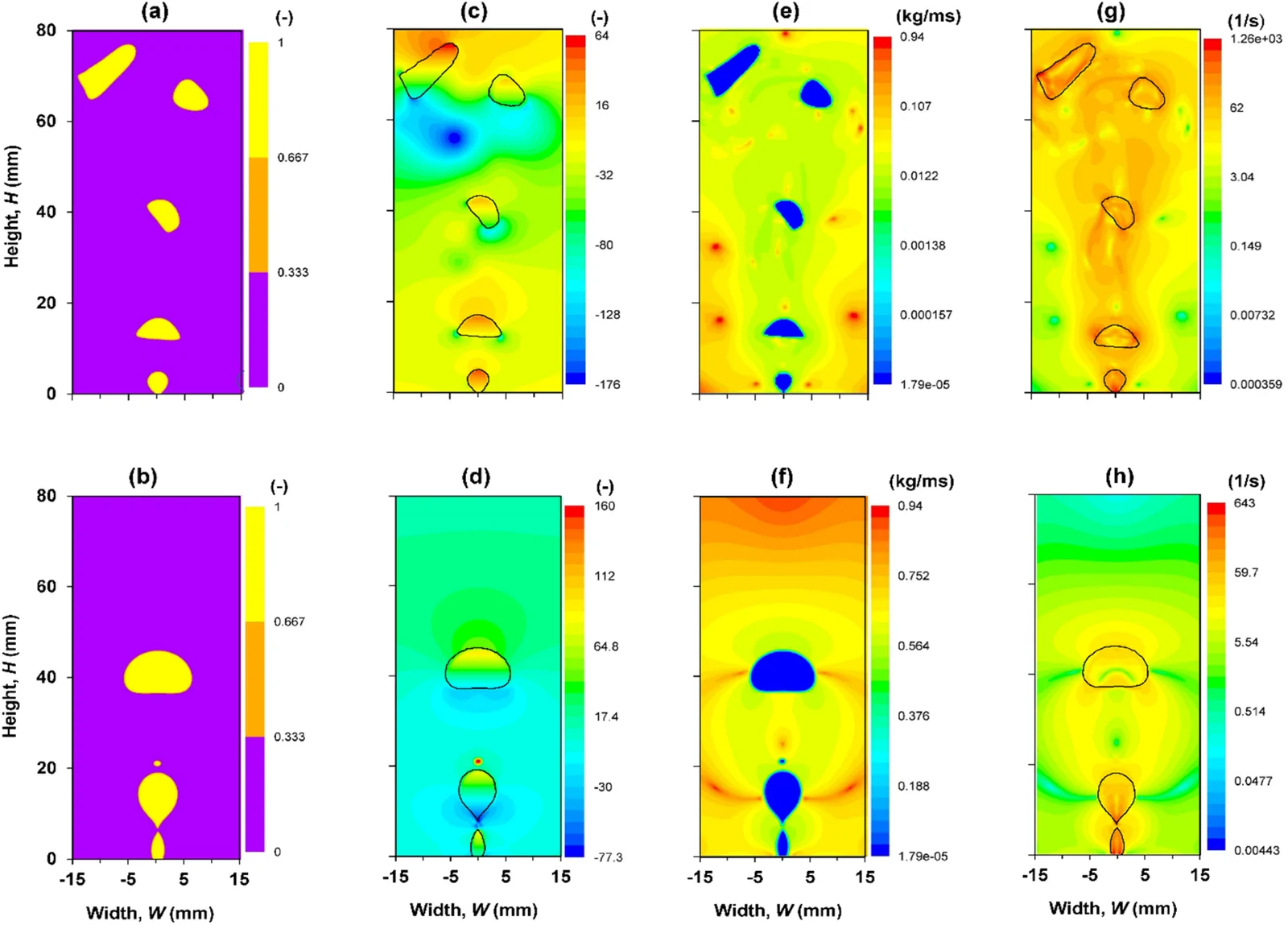
(a & b) Bubble shape, (c & d) pressure coefficient, (e & f) molecular viscosity, and (g & h) shear rate around bubbles in XG4 for *J*_G_ = 0.3 m s^−1^ (top row) *n* = 0.1, *Re* = 119.24, *Ar* = 1549.8, *Bo* = 0.136, *We* = 1.252, *Mo* = 1.1 × 10^−9^ and in XG6 for *J*_G_ = 0.3 m s^−1^ (bottom row) *n* = 0.9, *Re* = 0.62, *Ar* = 0.04, *Bo* = 0.136, *We* = 1.252, *Mo* = 1.5.

Fig. 8 shows the qualitative influence of the flow index on bubble formation by examining four leading characteristics. An increase in *n* notably affects bubble morphology. At a low *n* = 0.1, beginning from detachment, the bubble undergoes considerable deformation as it ascends within the solution (Fig. 8a). As *n* increases, the fluid exhibits elevated resistance to flow, resulting in slower bubble rise (Fig. 8b). Higher effective viscosity near the bubble minimizes deformation, maintaining a more spherical shape. At a high *n* = 0.9, the leading bubble assumes a flattened oblate ellipsoid shape.

Fig. 8c and d exhibit the pressure distribution changes surrounding the bubble due to changes in the flow index. A substantial rise in the pressure coefficient accompanies increased *n*, consistently displaying a higher-pressure coefficient above the bubble compared to beneath it. Fig. 8e and f show that molecular viscosity near the bubble is lower relative to its surroundings, which facilitates upward movement. In solutions with low *n* = 0.1, molecular viscosity appears irregularly distributed; in contrast, at a high *n* = 0.9, viscosity distribution intensifies along the far left and right sides of the bubble, with more viscosity observed beneath the bubble's core region. Regarding shear rate distribution, a reduction is evident in high *n*. In Fig. 8g, elevated shear rates occur at the bubble's lateral edges, while the third bubble exhibits a typical curved tail-shaped contour. In high *n*, the length of this tail diminishes, and both the left and right sides of the bubbles exhibit comparatively lower shear rates.

### 3.5. Comparison between the Carreau model and the power-law model

**Fig. 9:**
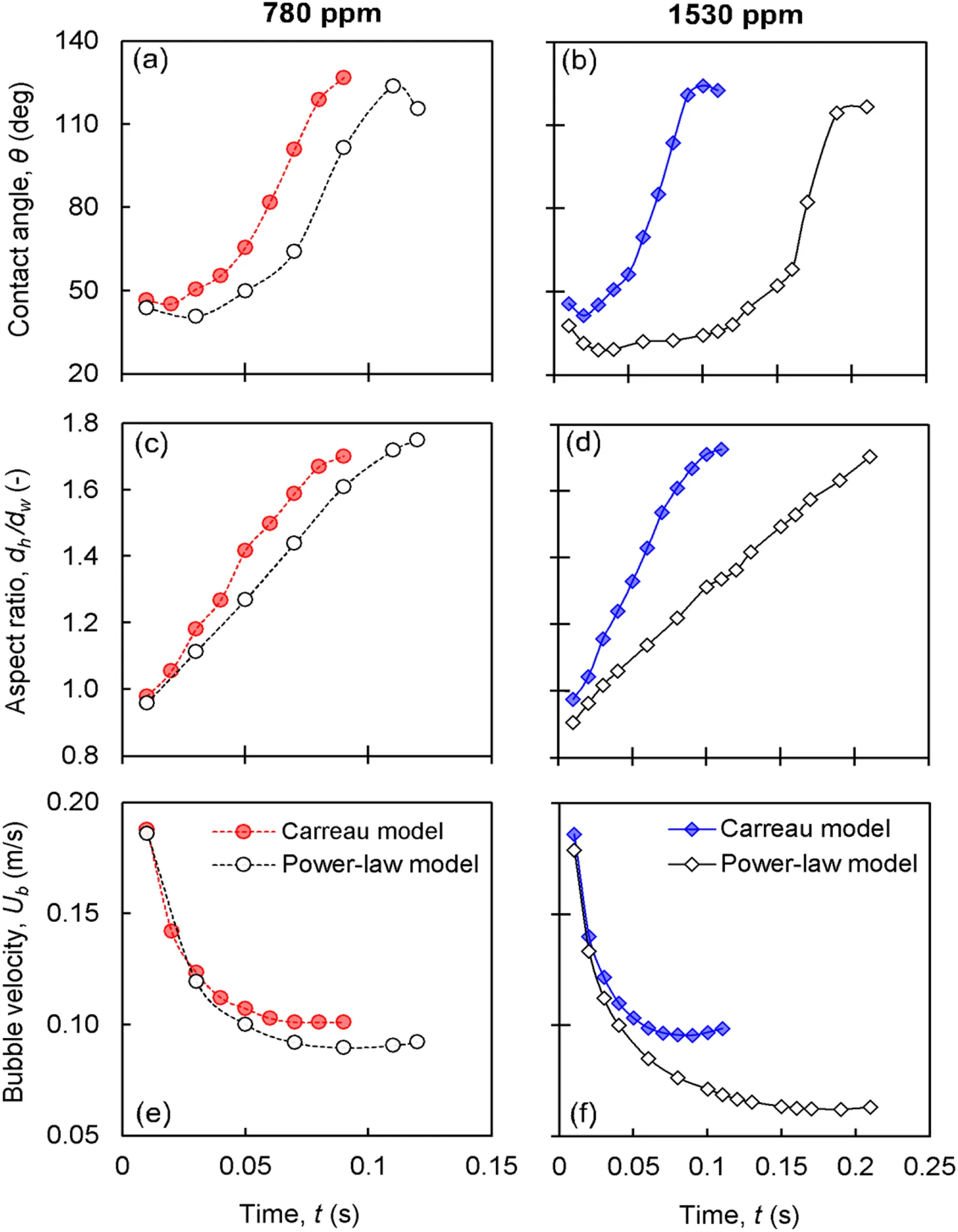
Influence of Carreau model and power-law (PL) model on bubble contact angle (a & b), aspect ratio (c & d), and velocity (e & f) for two different concentrations of 780 ppm (left column) and 1530 ppm (right column) at *J*_G_ = 0.3 m s^−1^.

Fig. 9 presents a comparison between the Carreau and Power-law (PL) models in predicting bubble formation, considering three salient features at an inlet velocity of 0.3 m s^−1^ for two different XG concentrations. The two models predicted different *θ* values at 0.3 m s^−1^ for both concentrations; however, the overall trend follows a similar profile in both models, and the PL model predicted lower values than the Carreau model. According to Fig. 9a, at 780 ppm, the pinch-off took place prior to 0.1 s and after 0.1 s for the Carreau and PL models, respectively. As presented in Fig. 9b, at increased XG concentrations, the difference among predicted values is noticeable. PL model predicted a prolonged expansion stage in bubble formation, whereas according to the Carreau model, there is an unsteady expansion phase during bubble formation. As predicted by the PL model, at higher XG concentrations, the bubble detachment took place after 0.2 seconds, which is 0.1 second longer than the detachment time predicted by the Carreau model.

Fig. 9c and d illustrate the differences between the two models based on the aspect ratio of bubbles. In the lower XG case, both models predicted a similar aspect ratio profile, which increases with time. At the pinch-off, the aspect ratio predicted by the Carreau model is 1.7, whereas PL model predicts a value over 1.7. Moreover, the slope of the aspect ratio curve is much steeper according to the Carreau model because the bubble shape changes more rapidly than that predicted by the other model. In the higher XG case, the changes in aspect ratio take place more slowly compared to the lower concentrations due to the presence of more viscous fluid near the bubble. The slope difference between the two curves predicted by the models increases significantly at a higher XG concentration.

The bubble velocity predicted by the two mentioned models is depicted in Fig. 9e and f for 780 ppm and 1530 ppm, respectively. In the lower XG case, the bubble velocity predicted by the two models at the beginning is around 0.19 m s^−1^, followed by a sharp decrease until 0.03 seconds. After that time, the velocity maintains a relatively constant value till the pinch-off. PL model predicted a lower final velocity than the Carreau model. A similar velocity reduction trend is noticed for higher XG, but due to the higher viscous force, which provides resistance in the bubble's upward motion, the velocity reduces gradually.

The differences between the two rheological descriptions are mainly in their behavior at low shear rates near the orifice, where the bubble first forms. The Carreau model constrains the viscosity between the zero-shear plateau, *µ*_0_, and the infinite-shear plateau, *µ*_∞_, so the predicted viscosity remains finite even as the shear rate approaches zero, whereas the PL model has no such lower bound and its viscosity grows without limit as the shear rate decreases toward zero. This unbounded low-shear viscosity in PL leads to higher predicted resistance to bubble growth immediately after gas injection, producing the longer pinch-off and detachment times and lower bubble velocities reported for the PL model relative to the Carreau model. The Carreau model is therefore expected to be the more physically realistic choice whenever the flow contains regions of very low shear rate, such as near a stagnant orifice or far from the rising bubble, whereas the PL model is most suitable over the fitted intermediate shear-rate range.

**Fig. 10:**
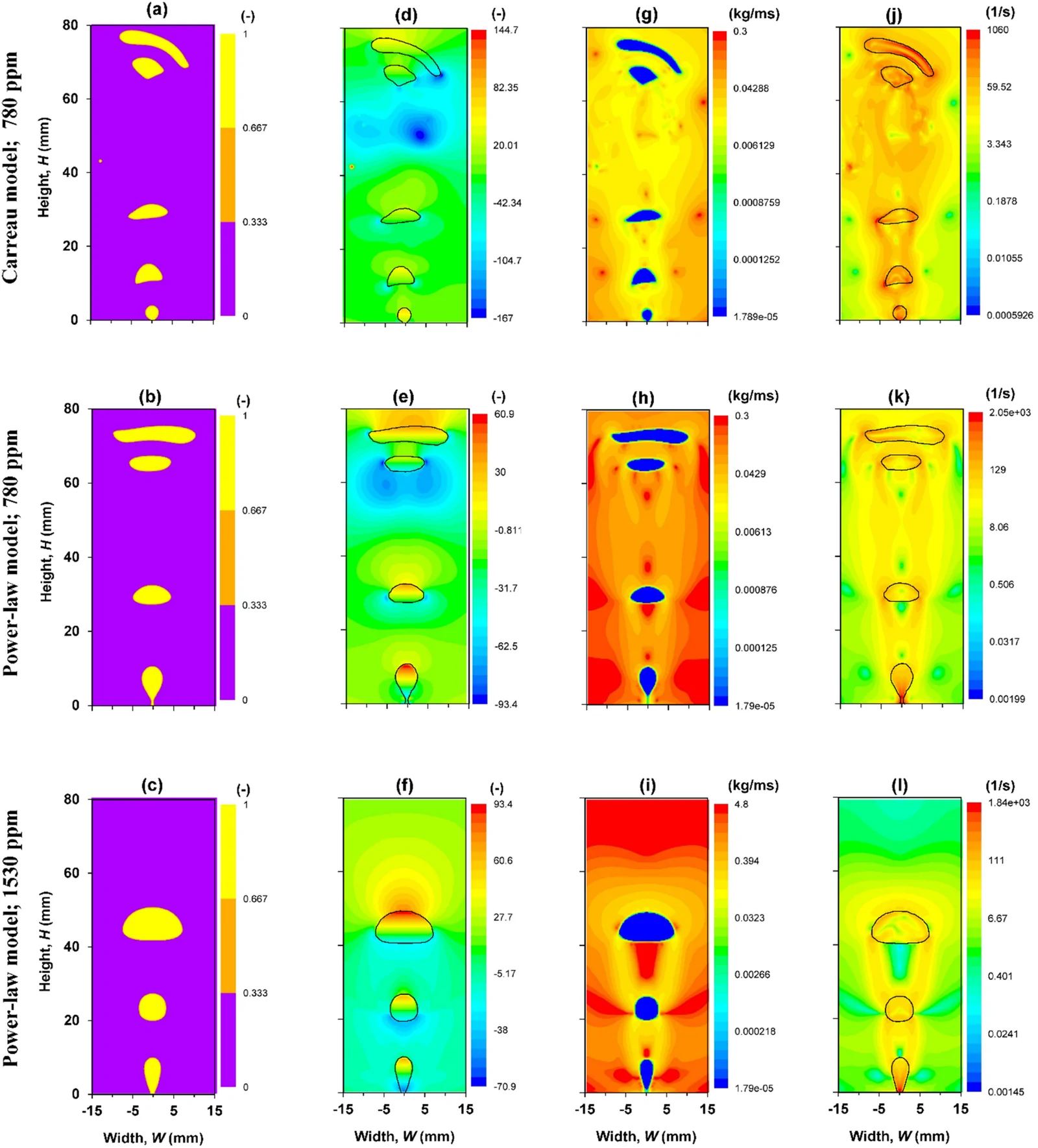
(a–c) Bubble shape, (d–f) pressure coefficient, (g–i) molecular viscosity, and (j–l) shear rate around the bubble for the Carreau model and power-law model at *J*_G_ = 0.3 m s^−1^.

The first and second rows of Fig. 10 show the comparative bubble rise characteristics predicted by the Carreau and power-law (PL) models for a 780 ppm XG at *J*_G_ = 0.3 m s^−1^. Fig. 10a and b show that both models anticipate a similar, flat shape for the leading bubble, with the bubbles reaching equivalent heights under both modeling approaches. However, the other bubbles' shapes vary in different models, and the shapes are less distorted according to PL model. Increasing the concentration decreases the number of bubbles as well as the distortion of bubbles, as shown in Fig. 10c, which is predicted by the PL model. The leading bubble is oblate ellipsoidal in a higher concentrated solution, whereas it is flat in a lower concentrated solution, as mentioned earlier.

The pressure coefficient predicted by PL model is significantly lower than the Carreau model, as shown in Fig. 10d and e. Both models anticipated similar trends of distribution, which is characterised by higher pressure in the upper section and lower pressure beneath the bubble. Furthermore, referring to Fig. 10f, PL model estimates higher pressure coefficients in the 1530 ppm XG solution. The reddish area in Fig. 10h is larger compared to Fig. 10g, which states that the molecular viscosity of the surrounding fluid estimated by PL model is higher. Both models reveal similar local pressure distributions near the bubble, though PL model reports lower values.

In relation to shear rate, Fig. 10k shows that for 780 ppm the PL model predicts a remarkably higher shear rate distribution than the Carreau model, manifested as straight, curved tails along the left and right edges of the bubble in the PL case. Conversely, the Carreau model displays a more irregular or chaotic shear rate distribution around the bubble (Fig. 10j). For XG = 1530 ppm, PL model predicts higher molecular viscosity and reduced shear rate distributions to the bubble. Specifically, viscosity is raised on the lateral sides of the bubble, while the shear rate is substantially weakened, as represented in Fig. 10i and l, respectively.

## 4. Conclusion

The dynamic characteristics of a bubble ascending through a non-Newtonian xanthan gum solution were numerically simulated using the VOF method. The simulation was evaluated against three modified bubble–size correlations, and the lowest error range (8.93–0.31%) was obtained against eqn (13); this correlation-based validation should be interpreted within the limitations of the effective viscosity approximation. The main findings of the study are -

• Bubbles' pinch-off duration and the aspect ratio decreased significantly with the increase in air velocity for all three different XG concentrations.

• With the increase of the flow index, a reduction in the contact angle and the bubble velocity is noticed; on the contrary, the aspect ratio, the duration of the expansion stage, and pinch-off increased significantly. Apart from these, the bubbles experience less deformation at higher XG concentrations.

• The power-law model predicts a longer bubble pinch-off time than the Carreau model at every XG concentration, and at higher XG concentrations. Hence, the power-law model shows a prolonged expansion stage during bubble formation. During pinch-off, the power-law model predicts a higher aspect ratio and a lower bubble velocity.

• At 780 ppm, both the power-law model and the Carreau model predicted an identical gradual bubble deformation from the orifice to the top of the column, whereas according to the power-law model, at higher XG concentration, the leading bubble in the column takes the oblate ellipsoidal shape.

• The pressure coefficient around the bubble is lower, but the shear rate is higher according to the power-law model.

The current model is limited to isolated bubbles in a two-dimensional column and does not include bubble–bubble interactions, coalescence, breakup, surfactant contamination, or fully three-dimensional path instabilities. The XG concentration range is also constrained to the available rheological data; so, extrapolation outside this range requires further validation and requires future work to investigate the above phenomena. From an engineering perspective, the predicted sensitivity of detachment diameter, pinch-off time, and rising velocity to XG concentration and gas velocity assists in guiding separator design, sparger selection, and process-control strategies.

## Conflicts of interest

There are no conflicts to declare.

## Abbreviations

*D*
: Computational domain width (mm)
*d*_b_
: Equivalent bubble diameter (mm)

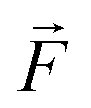

Volume Force (N)
*F*_b_
: Buoyancy force, (N)
*F*_in_
: Inertia force, (N)
*F*_vis_
: Viscous force, (N)
*F*_suf_
: Surface tension force, (N)

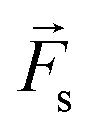

Body force, (N)
*F*_d_
: Viscous drag force, (N)
*g*
: Acceleration due to gravity, (m s^−2^)
*H*
: Computational domain height (mm)
*J*_g_
: Air velocity, (m s^−1^)
*K*
: Consistency coefficient (−)
*n*
: Flow index of shear-thinning fluid (−)
*P*
: Liquid pressure, (N m^−2^)

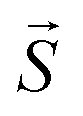

Strain rate tensor (s^−1^)
*t*
: Time, (s)
*U*_b_
: Bubble rising velocity (m s^−1^)

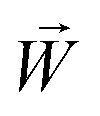

Liquid velocity (m s^−1^)
*σ*
: Surface tension (N m^−1^)
*µ*_L_
: Liquid viscosity, (Pa s)
*ρ*_L_
: Liquid density, (kg m^−3^)
*γ̇*
: Local shear rate (s^−1^)
*θ*
: Contact angle (deg)
*∅*_L_
: Volume fraction function (−)
*Ar*
: Archimedes Number (−)
*Bo*
: Bond Number (−)
*Mo*
: Morton Number (−)
*Re*
: Reynolds Number (−)
*We*
: Weber Number (−)
*Ca*
: Capillary number (−)
XG: Xanthan Gum (ppm)

## Data Availability

The data generated during and/or analysed during the current study are available from the corresponding author on reasonable request.
